# Refractory severe type B insulin resistance treated with daratumumab

**DOI:** 10.1210/jcemcr/luag145

**Published:** 2026-05-27

**Authors:** Samhitha Munugoti, Maneesh Gaddam, Natasha Rasool, Pavel Shevtsov, Paramarajan Piranavan, Philip A Kern

**Affiliations:** Division of Endocrinology, Department of Internal Medicine, University of Kentucky, Lexington, KY 40536, USA; Division of Pulmonary and Critical Care Medicine, Appalachian Regional Healthcare, Hazard, KY 41701, USA; Division of Rheumatology, Department of Internal Medicine, University of Kentucky, Lexington, KY 40536, USA; Division of Rheumatology, Department of Internal Medicine, University of Kentucky, Lexington, KY 40536, USA; Division of Rheumatology, Department of Internal Medicine, University of Kentucky, Lexington, KY 40536, USA; Division of Endocrinology, Department of Internal Medicine, University of Kentucky, Lexington, KY 40536, USA

**Keywords:** insulin receptor autoantibody, systemic lupus erythematosus, CD19, CD38, obinutuzumab

## Abstract

Type B insulin resistance syndrome (TBIRS) is a rare autoimmune condition characterized by insulin receptor autoantibodies, often in association with systemic lupus erythematosus (SLE). It can cause extreme insulin resistance, with fewer than 200 cases described worldwide. A 24-year-old man with no prior autoimmune history presented with diabetic ketoacidosis and severe insulin resistance, requiring up to 215 000 units of insulin daily. Laboratory studies revealed high-titer antinuclear antibodies, hypocomplementemia, lupus-specific autoantibodies, and mesangial proliferative lupus nephritis, confirming new-onset SLE. His management was complicated by latent tuberculosis, neutropenia, and cyclophosphamide-induced hearing loss. Despite intensive therapy with corticosteroids, rituximab, cyclophosphamide, obinutuzumab, plasmapheresis, and intravenous immunoglobulin, he remained profoundly insulin resistant, requiring very high daily insulin doses, with persistently elevated insulin receptor antibody titers. Initiation of daratumumab, a CD38 monoclonal antibody, resulted in marked clinical improvement, with progressive decline in insulin requirements and eventual discontinuation of insulin 17 months after presentation. He achieved durable remission with normalization of hemoglobin A1c. This unusually severe case of TBIRS highlights the limitations of conventional B-cell–directed therapies, where long-lived plasma cells sustain pathogenic autoantibodies. Plasma cell–targeted strategies such as daratumumab may represent an effective therapeutic option in refractory cases.

## Introduction

Type B insulin resistance syndrome (TBIRS) is a rare autoimmune condition characterized by the presence of autoantibodies against the insulin receptor, resulting in severe insulin resistance and extreme hyperglycemia. It is most often associated with systemic autoimmune diseases, particularly systemic lupus erythematosus (SLE) [1, 2]. Due to its rarity, fewer than 200 cases have been described globally. In one review TBIRS was more common in women and African Americans [3]. The autoantibody epitopes can vary, and patients can present with severe insulin resistance or occasionally hypoglycemia [4, 5].

Here, we present a striking case of TBIRS in a young male with no prior history of autoimmune disease, who developed life-threatening insulin resistance requiring insulin doses as high as 215 000 units per day. His clinical course was further complicated by latent tuberculosis, neutropenia, and he was refractory to many immune modifying regimens, but finally responded to daratumumab, an anti-CD38 monoclonal antibody used for the treatment of multiple myeloma. The case highlights a severe case of TBIRS with management complexities and the need for a flexible, individualized immunotherapeutic approach.

## Case presentation

**Figure 1:**
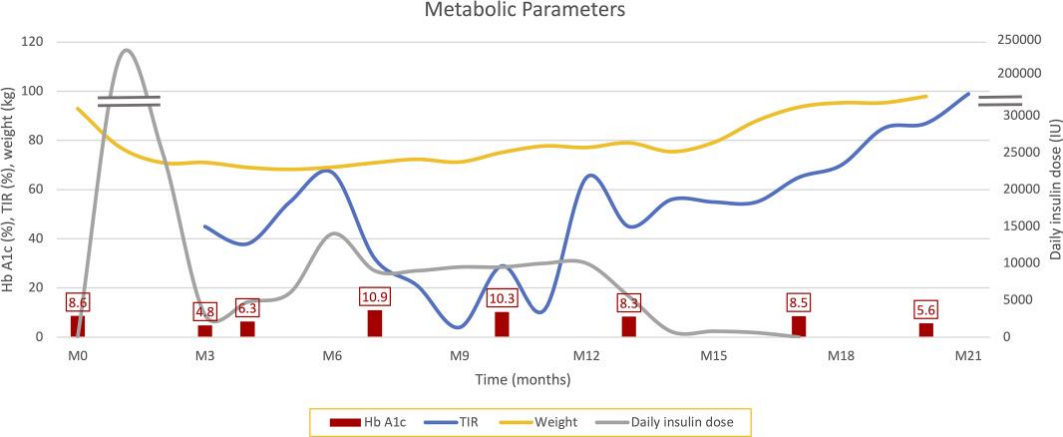
Trends in metabolic parameters over time. HbA1c, time in range (TIR, percentage of time with glucose of 70 to 180 mg/dL), body weight (kg) and daily insulin dose (Units/day) are shown over time starting with the patients initial presentation.

A 24-year-old male with no known prior medical history presented to the emergency department with 1 week of hematuria, fatigue, polyuria, polydipsia and weight loss. The patient weighed 92 kg with a body mass index of 28 kg/m^2^ (reference range: 18.5 to 24.9 kg/m^2^) and physical examination was notable for bilateral axillary acanthosis nigricans. Laboratory evaluation revealed a blood glucose level of 497 mg/dL (SI: 27.6 mmol/L) (reference range <100 mg/dL [SI: 5.6 mmol/L]), an anion gap of 13 mEq/L (reference range 3 to 11 mEq/L), and an elevated beta-hydroxybutyrate level of (SI) 3.49 mmol/L (conventional: 36.3 mg/dL; reference range <.6 mmol/L[SI]; <6.2 mg/dL [conventional]). He was diagnosed with diabetic ketoacidosis (DKA) and admitted to the intensive care unit (ICU), where an insulin drip was initiated. There were no signs of infection, the patient had no previous diagnosis of diabetes mellitus or family history, and his hemoglobin A1c (HbA1c) was 8.6% (SI: 70 mmol/mol) (reference range <5.7% [SI: <48 mmol/mol]). Initially, he required up to 50 units of insulin per day and he was transitioned to a basal-bolus insulin regimen. However, he then redeveloped DKA with very high glucose levels and required high doses of insulin, exceeding 70 units per hour. The overall course of metabolic parameters is shown in Fig. 1.

## Diagnostic assessment

The patient also complained of arthralgias, and with the escalating insulin requirements a workup for SLE was initiated. The patient tested positive for antinuclear antibodies (ANA) with a speckled pattern at a titer >1:2560, had marked hypocomplementemia (low C3 and C4 levels), and was positive for anti-Smith, anti-double-stranded DNA (anti-dsDNA), and anti-RNP antibodies. Anti-Ro, anti-La, and antiphospholipid (APL) antibodies were negative. A kidney biopsy revealed mesangial proliferative lupus nephritis. Despite the presence of anti-RNP antibodies, the patient did not demonstrate sufficient clinical features for mixed connective tissue disease.

There was no evidence of Cushing syndrome or prior steroid use, no history of rare monogenic insulin resistance, islet cell antibodies were negative and there were no clinical features of lipodystrophy.

**Figure 2:**
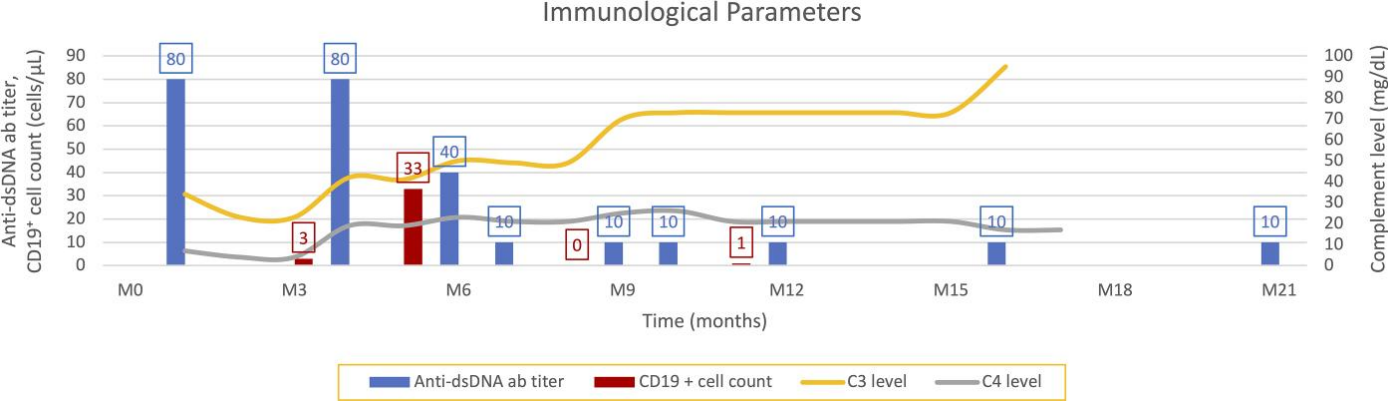
Trends in immunologic parameters over time. The changes in anti-dsDNA ab titer, CD19 + cell counts, C3 and C4 levels are shown beginning with the patients initial presentation.

TBIRS was suspected in the setting of SLE, and an elevated C-peptide (18.5 ng/mL, SI: 6.13 nmol/L) (reference range 0.5 to 2.0 ng/mL [SI: 0.17 to 0.66 nmol/:L]) and a normal adiponectin level was demonstrated. The patients blood glucose levels remained > 600 mg/dL (SI: 33.3 mmol/L), insulin drip requirements exceeded 100 units per hour. Serum from the patient along with control samples were sent to Dr. Lutz Schomburg (Berlin, Germany) for measurement of insulin receptor antibody [6] and were strikingly positive (Table 1). The overall course of immunologic parameters is shown in Fig. 2.

**Table 1:** Insulin receptor antibody levels during treatment

Date*^a^*	Insulin dose	Average Glucose	Insulin receptor antibody binding index (BI)*^b^*			
			Positive control	Negative control	Control subjects	Patient
Month 2	216 000 U/day	500 mg/dL (27.8 mmol/L)	22.6 BI	1.9 BI	1.4 to 5.0 BI	283.1 BI
Month 10	10 000 U/day	269 mg/dL (14.9 mmol/L)	224.4 BI	1.0 BI	1.0 to 1.3 BI	145.5 BI

^*a*^Date is relative to the initial presentation (see Fig. 1).

^*b*^BI. The binding index indicates the binding of serum antibodies to insulin receptor normalized to control sera. The positive and negative control samples are standards used in the assay. Samples from 4 control subjects without diabetes were analyzed alongside the patients sample.

In anticipation of anti-immune therapy, a QuantiFERON-TB Gold test was obtained, which was positive. Chest X-ray and tuberculosis cultures were negative and rifampin was begun to prevent reactivation of latent tuberculosis.

## Treatment

Treatment of the TBIRS was initiated with rituximab, cyclophosphamide, and pulse-dose corticosteroids following previously published guidelines [7]. The patient was already hyperglycemic, with blood glucose over 500 mg/dL (SI: 27.8 mmol/L) in spite of over 8000 U insulin/day to prevent DKA, and there was concern that the treatment regimen, which included high dose steroids, would worsen the insulin resistance. A high concentration insulin drip (6000 units reconstituted in 250 mL) was initiated, along with 2500 units three times daily subcutaneously using U-500 insulin, timed with the first dose of steroids. Despite these interventions, the patients insulin requirements escalated dramatically, peaking at 11 000 units per hour, along with 4500 units per meal, totaling up to 215 000 units of insulin daily. Pulse-dose steroids were discontinued after the second dose, and plasmapheresis was initiated, which led to a notable decrease in insulin requirements. After three sessions of plasmapheresis the patient was weaned off the insulin drip, though he still required approximately 2000 to 4000 units of subcutaneous insulin with meals. Pulse-dose steroids were reinitiated to complete cycle 1 of the above described treatment protocol, and a total of six plasmapheresis sessions were performed.

The patient was hospitalized for 90 days and initially treated with hydroxychloroquine, oral cyclophosphamide, rituximab, dexamethasone, pulse-dose corticosteroids, and two cycles of plasmapheresis (five additional sessions in the second cycle). Neutropenia and low-grade fevers developed within one week of oral cyclophosphamide (at both 100 and 50 mg), prompting discontinuation, temporary broad-spectrum antibiotics, and later a permanent switch to intravenous (IV) therapy. He also developed cyclophosphamide-associated hearing loss. Rituximab 2 g was administered but failed to achieve adequate B-cell depletion, with CD19 + levels at 8.8%. Insulin requirements improved somewhat, allowing discharge to outpatient care with a continuous glucose monitor (CGM), metformin, and U-500 insulin at 8000 to 12 000 U/day. At that time, he weighed 67 kg, was very weak from muscle loss, and had significant alopecia and his acanthosis nigricans was much worse with diffuse patchy skin darkening all over.

On outpatient follow-up, obinutuzumab 2 g was initiated, leading to complete CD19 + depletion. Once neutropenia resolved, IV cyclophosphamide 500 mg monthly was started, later escalated to 750 mg every 2 to 4 weeks for eight additional cycles (total dose 8 g over nine months). Despite therapy, the patients insulin requirements remained high (6000–12 000 U/day depending on immunosuppressive regimen), with persistent hyperglycemia, florid acanthosis nigricans and fatigue. After 14 months, his weight had increased to 76 kg. Repeat insulin receptor antibody titers were improved but remained markedly elevated (Table 1).

**Figure 3:**
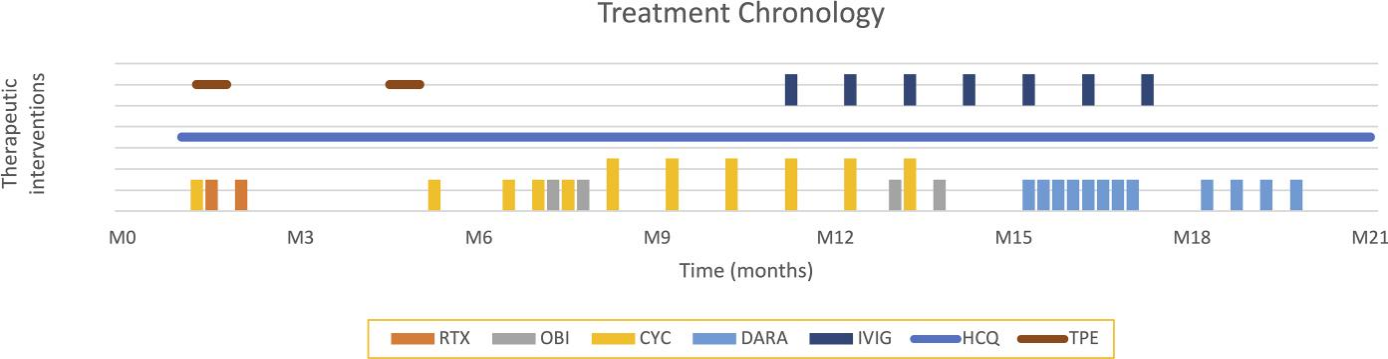
Chronology if immunomodulatory and adjunctive therapies over time. The vertical bars represent discrete intravenous infusions administered at specific time point. Horizontal bards represent continuous therapies, including daily oral medications and defined periods of serial therapeutic plasma exchange (TPE). CYC, cyclophosphamide; DARA, daratumumab; HCQ, hydroxychloroquine; IVIG, intravenous immunoglobulin; ObI, obinutuzumab; RTX, rituximab.

Because of refractory disease after two cycles of obinutuzumab and extended IV cyclophosphamide, intravenous immunoglobulin (IVIG) 2 g/kg was initiated but was ineffective after four cycles. Therapy was then transitioned to plasma cell–directed treatment with daratumumab. After nine doses, the patient achieved remission and was able to discontinue insulin. Figure 3 illustrates the overall course of treatment over time.

## Outcome and follow-up

Upon receiving weekly daratumumab infusions, his insulin requirements decreased, and after receiving eight doses of daratumumab, insulin therapy was successfully discontinued 17 months after his initial presentation. The patient gained back the weight he had lost, his acanthosis faded and he was able to resume employment. Nineteen months following his presentation, the patients HbA1c was 5.6%, on no insulin or metformin and the CGM was discontinued.

## Discussion

This case of TBIRS is distinguished by several unique and clinically significant features. Unlike most reported cases, our patient had no prior symptoms of SLE, and lupus was only uncovered during the evaluation for autoimmune insulin resistance. He presented with extreme hyperglycemia, requiring up to 215 000 units of insulin daily, one of the highest documented requirements in the literature. The initiation of immunosuppression was further complicated by a concurrent diagnosis of latent tuberculosis, necessitating prophylactic therapy with rifampin before steroid and cytotoxic administration. Additionally, the patient experienced numerous complications, including prolonged neutropenia and neutropenic fever, required temporary cessation of immunosuppression, sensorineural hearing loss and hair loss.

The underlying mechanism of TBIRS involves IgG autoantibodies targeting the insulin receptor, most often its α-subunit [4]. These autoantibodies may act as antagonists by blocking insulin binding or promoting receptor degradation, both of which result in severe insulin resistance and marked hyperglycemia [4]. In some cases, they may behave as partial agonists, stimulating the receptor inappropriately and causing episodes of hypoglycemia [5]. When functioning as inhibitors, these antibodies disrupt the PI3K/Akt signaling cascade downstream of the insulin receptor, thereby impairing glucose uptake and rendering both endogenous and administered insulin ineffective [8]. In our case, normal adiponectin levels despite severe hyperinsulinemia, absence of ZnT8, GAD65, IAA, ICA autoantibodies, and high C-peptide levels raised early suspicion for TBIRS, which was later confirmed by positive anti-insulin receptor antibody titers. In our patient, the rapid resolution of hyperglycemia with minimal insulin requirement following plasmapheresis suggests that the antibodies were likely inhibitory to the insulin receptor rather than receptor destructive. While the exact mechanism triggering autoantibody development is unclear, molecular mimicry in the setting of systemic autoimmune activation is a proposed mechanism. The overlap with connective tissue diseases suggests shared immunogenetic susceptibility. Consistent with cases reported in the literature, our patient developed insulin receptor antibodies in the context of SLE, which met standard SLE classification criteria [9].

Many patients with TBIRS respond well to a previously published protocol that includes rituximab, corticosteroids, and cyclophosphamide [7] and go into remission with discontinuation of insulin therapy after 4 to 6 months, whereas this patient required insulin for 17 months, mostly very high dose insulin which was challenging for both in- and outpatient delivery.

In this patient, conventional B-cell and lymphocyte-directed therapies failed because long-lived plasma cells remained a persistent reservoir of autoantibody production. Antibodies such as anti-Smith, RNP, Ro, and La in SLE derive from long-lived plasma cells, whereas anti-DNA antibodies typically arise from plasmablasts and fluctuate with disease activity [10]. Anti-proliferative agents like mycophenolate and azathioprine suppress plasmablast-driven antibodies by targeting lymphocytes reliant on the de novo purine pathway but leave terminally differentiated plasma cells unaffected [10]. Likewise, B-cell depletion with rituximab or obinutuzumab did not control disease activity, as plasma cells lack CD20 expression. Even obinutuzumab, a more potent CD20 agent effective in lupus nephritis trials [11] and TBIRS [3], failed to improve the patients condition. Cyclophosphamide was limited by toxicity and incomplete plasma cell depletion, while IVIG offered no clinical benefit. This course strongly suggested that pathogenic insulin receptor antibodies in this case were generated by long-lived plasma cells.

This case highlights the critical need for plasma cell–directed therapy in refractory autoimmune disease. Plasma cells, uniquely adapted for continuous immunoglobulin synthesis, depend heavily on protein-handling systems such as proteasome function and express surface markers like CD38, which are amenable to targeting. Therapies including proteasome inhibitors, BCMA-directed agents, and monoclonal antibodies against SLAMF7 or CD38 exploit this biology to eliminate plasma cells while sparing most other immune subsets [12, 13]. In this patient, daratumumab, a CD38 monoclonal antibody with a favorable safety profile compared with proteasome inhibitors, was selected for tolerability and anecdotal efficacy in lupus nephritis [12, 14-16], although this drug is mostly used for multiple myeloma. Crucially, daratumumab achieved what prior regimens could not: durable remission with elimination of insulin dependence, directly demonstrating the central role of plasma cells in driving disease. This case underscores the limits of conventional immunosuppression where plasma cells sustain autoantibody production and illustrates how plasma cell depletion provides a rational and effective strategy in otherwise refractory autoimmunity.

This is, to our knowledge, the first reported case in the literature of a patient with TBIRS requiring three different monoclonal antibody regimens to achieve remission and also the first case of the use of daratumumab.

## Learning points

- Type B insulin resistance syndrome (TBIRS) is rare and characterized by autoantibodies to the insulin receptor, usually in the context of other autoimmune diseases.
- Insulin resistance is from the autoantibody and can be severe, requiring high doses of insulin.
- Treatment involves chemotherapy directed against antibody producing B cells and plasma cells.
- Collaboration among different specialties, particularly endocrinology, rheumatology and chemotherapy experts to adequately treat this complex problem.

## Data Availability

Original data generated and analyzed during this study are included in this published article.
